# Pollinator cognition in a plant network

**DOI:** 10.1098/rsbl.2025.0044

**Published:** 2025-05-28

**Authors:** Patricia L. Jones, Eric M. Diaz, Neena E. Goldthwaite, Hannah T. Scotch, Sejal V. Prachand, Eva R. Ahn

**Affiliations:** ^1^ Department of Biology, Bowdoin College, Brunswick, ME, USA

**Keywords:** associative conditioning, floral reflectance, generalist, learning, plant–pollinator network, specialist

## Abstract

Cognitive abilities evolve within the context of ecological communities. Honeybees and bumblebees have become model systems for cognitive ecology, but pollination is performed by a diverse group of insects under similar pressures to forage efficiently in a mixed floral community. We studied the colour learning abilities of six species of Hymenoptera (two eusocial bumblebees, a cuckoo bumblebee, two wasps and a leaf-cutter bee) within the context of an island plant community. We used records of insect visits to flowers in the field to determine the index of specialization of each species in the island plant–pollinator network, and measured the spectral reflectance of the flowers they visit. Species with higher specialization indices in our plant–pollinator network made a larger proportion of correct choices in a colour learning task than more generalist species. The more generalist species also visited a group of flowers more similar to each other in hymenopteran colour vision space. These results indicate that better colour learning abilities may enable insects to forage on plants of different colours, whereas more generalist insects are visiting flowers that are similar in colour, and therefore are less reliant on repeated colour learning to forage efficiently.

## 1. Introduction

Flowers have multimodal traits that pollinators must detect and process using their sensory systems [1]. The social bees have been well-studied for their abilities to learn floral traits, including colours [2], scents [3], patterns [4] and even electric fields [5]. The ability to learn and remember floral information may enable flower visitors to forage more efficiently on rewarding flower types [6,7] (although see [8]). If learning results in an increase in floral constancy, or the tendency to visit one flower type amidst multiple options, this may in turn enhance plant pollination by increasing the ratio of conspecific to heterospecific pollen deposition [9]. The decisions bees make, and the learning abilities that influence those decisions, should shape and be shaped by the floral community in which they forage. Bees are not the only flower visitors within a plant community; there are other pollinators who are subject to the same selective pressures to choose between floral options, but whose learning capabilities are less well studied. These pollinators often have different sensory systems, and likely different sensory biases for floral stimuli [2,4,10,11]. Which flowers a pollinator decides to visit is influenced by a combination of sensory perception, biases and learning abilities [12]. There has been a recent surge in research to understand how floral traits including colour [13–17], scent [14,18], and phenology [14,15,19,20], structure plant–pollinator networks and impact degrees of specialization by flower visitors. For example, overlaps in flowering colour [13,15] or scent [14] can increase pollinator sharing between plants and, therefore, generalization at the pollinator species level. What is missing from this recent work on network dynamics and specialization is the role of pollinator perception and learning in floral visits.

Pollinator learning of plant traits likely influences ecological specialization, but in complex ways. Theoretical predictions [21] include that extreme specialists may require little learning abilities as floral preference could be dictated by genetic mechanisms. Generalists, because they visit a variety of plant species that change in space and time, should then be better learners [22]. At the same time, extreme generalists might visit whatever flowers are available with little role of learning in foraging choices. The highest learning abilities might then be expected in species with intermediate levels of specialization [21]. This theory is complicated by the fact that within many pollinator species considered at the species level to be generalists, there is high individual specialization [23,24]. For example, DNA identification of pollen loads has demonstrated that within generalist pollinator species individuals carry pollen loads that indicate specialization on particular plants [25]. The same phenomenon can be true in herbivores, where generalist species may be comprised of specialized individuals [26]. This individual specialization may be a product of individual learning of floral phenotypes. To further complicate analyses of specialization in pollinators, insects could differ in their levels of plant specialization when pollen versus nectar foraging. Additionally, the local community context can have strong effects on pollinator niche breadth, making it difficult to transfer assessments of levels of specialization between studies [23].

The perception of floral traits by insect sensory systems and associative learning abilities likely interact to shape plant–pollinator network structure, but they are rarely studied in combination. To understand how these factors interact, we examined the plant–pollinator network of a temperate North American island community and measured floral spectral reflectance to assess how one important signal, flower colour, is perceived via insect sensory systems. We used our plant–pollinator network structure to assign insect species a level of specialization (*d*′) and then tested whether that degree of specialization was associated with variation in the colours of flowers that insects are visiting, e.g. whether insects that are assigned as more generalized in our network visit a group of flowers that are more widely varied in colour than more specialized insects. We then captured individuals from insect species that were common in our network and tested their associative learning abilities for blue and yellow stimuli using an established protocol. We predicted that colour learning abilities would be correlated with levels of network specialization such that more specialized insects would show better performance on an associative learning task as supported by previous work [27]. An alternative prediction is that generalist insects show better associative colour learning, which might be expected if generalists are visiting flowers of a wider range of different colours. Examining network specialization, floral reflectance and associative colour learning abilities in the same plant community enables novel insight into how these factors interact to shape insect decision-making and community dynamics.

## 2. Methods

### (a) Plant–pollinator network

This study was conducted at the Bowdoin Scientific Station on Kent Island, an 80 ha island in New Brunswick, Canada. Kent Island is 32 km from the closest mainland in Cutler, ME, 5 km from the closest larger island of Whitehead, NB and 8 km from the larger island of Grand Manan, NB. In June and July of 2019, 2022 and 2023 one to two researchers collected insects when they were observed visiting flowering plants across the island. Across all three collection years, insects were identified to the lowest taxonomic group possible using keys and by posting photos on iNaturalist. Tissues from a subset of 95 insects from 2022 were submitted to the Canadian Center for DNA barcoding for identification using the CO1 barcoding region (BOLD Project ‘Identifying Insect Flower Visitors in Plant Pollinator Network on Kent Island, New Brunswick Canada’).

We used the ‘bipartite’ package [28] in R to visualize the plant–pollinator network and calculate indices of specialization of the insect species. To assess how representative a network is from a given year, we compared the network data between years with a Jaccard similarity index for interactions using the ‘vegdist’ command in the R package ‘vegan’ [29]. We evaluated sampling completeness with a Chao 2 estimator [30,31], using the ‘specpool’ command in the ‘vegan’ package. To determine specialization indices for pollinator species in the network (*d*′) [32], we used the ‘dfun’ command in the ‘bipartite’ package.

### (b) Floral reflectance spectra

In 2022 and 2023, we measured the reflectance spectra of collected flowers using an Ocean Optics Flame miniature spectrometer with a DH-2000 BAL UV–VIS–NIR light source and PTFE diffuse reflectance standard (Ocean Optics, Orlando, FL, USA). For each plant species, we measured floral reflectance from three different representative specimens and used the average reflectance of each wavelength from the three specimens for further analysis. We plotted our stimuli in the hexagonal colour vision space of the honeybee *Apis mellifera* [33] using the R package ‘pavo 2’ [34]. We used the honeybee spectral sensitivities for our visual model because while there are published spectral sensitivities for some bumblebee species [35,36], they do not include the species we tested, and there are no spectral sensitivities published for our wasps or leaf-cutter bees. Hymenopteran colour vision is considered quite conserved [37–39], so we felt the most appropriate system to use was the well-studied honeybee. For each pollinator species we tested in our behaviour experiment we created a vector of the pairwise chromaticity distances between the flowers they visited in our network. We used a linear model to examine the relationship between the *d*′ (index of specialization) of the species and the chromaticity distances between visited flowers. To compare how similar the two stimuli used in our colour learning experiment were to flower colours, we created vectors of pairwise chromaticity distances between a test stimulus and the flowers in the community and compared the vectors using a two-sample *t*‐test.

### (c) Colour learning experiment

We examined how abilities to associate colours with rewards relates to the flower colours insects are visiting in the field. In 2022 and 2023, we tested the associative colour learning abilities of six hymenopteran species: *Bombus flavidus* (*n* = 15), *Bombus sandersoni* (*n* = 87), *Bombus ternarius* (*n* = 19), *Dolichovespula arenaria* (*n* = 40), *Dolichovespula norvegicoides* (*n* = 44) and *Megachile melanophaea* (*n* = 13). Insects collected on flowers in the field were returned to the laboratory and transferred into clean 50 ml tubes with a modified cap into which we inserted transparency paper with two holes ~1.5 cm apart. Insects were allowed to acclimate to the new tubes for 30−90 min before testing. Colour learning was tested using Free-Moving Proboscis Extension Response (FMPER) [40] following established protocols [41]. Insects were trained to associate either blue or yellow strips of paper (conditioned stimulus, CS) with a 50% sucrose solution reward (unconditioned stimulus, US). Papers were Neenah Astrobrights ‘Sunburst Yellow’ and ‘Blast-Off Blue’ (Neenah Paper, Atlanta, GA, USA). Treatments were balanced such that for each species an equal number of individuals received blue as the rewarded CS (CS+) and yellow as the CS+. Each insect received two training trials and five choice trials. For the training trials, we dipped the designated rewarded colour strip in 50% sucrose solution (CS+) and inserted it into one side of the cap (we alternated the offered side between individuals). We allowed insects to drink from the paper for a maximum of 3 s and then removed the strip and inserted the alternative colour strip, which had been dipped in water (CS−) inserted into the opposite side of the cap. Insects were allowed to drink the water from the CS− for 3 s, and then it was removed, or if insects did not drink we removed the strip after the insect touched it with an antenna. Insects had to make antennal contact with the strip to continue. For the second training trial, we switched the sides of the presentation of the two stimuli. There was a ~10 min interval between each training trial and between training trials and the choice trials.

After the two training trials, we gave each insect five subsequent rewarded choice trials. For choice trials, we simultaneously inserted the CS+ (with sucrose) and CS− (with water) and the first strip the insect touched with its antennae or proboscis was recorded as its choice. The insect was allowed to drink for 3 s before the strip was removed. The other strip was left in the tube until the insect drank from it for 3 s or touched it with an antenna. We repeated this for a total of five times, alternating the colour presentation sides each time with 10 min between each trial. To prevent re-testing the same insect, we placed a dot of fluorescent pink acrylic paint (Sargent Art, Hazleton, PA, USA) on the thorax of each insect and released them back into the field. We analysed the performance of insects using binomial generalized linear mixed-effect models (GLMM) in the ‘lme4’ package [42] in R with a choice of the CS+ as 1 and a choice of the CS− as 0. Treatment (whether the CS+ was blue or yellow), insect species, the interaction between treatment and insect species, trial number and the interaction between trial number and species were included as fixed effects and individual insect IDs were included as a random effect.

## 3. Results

### (a) Plant–pollinator network

The plant–pollinator network had 3718 observations of 145 different types of flower visitors (identified to the lowest taxonomic group feasible) on 74 plant species. Flies were the most common visitors in our dataset at 44.5%, followed by bees 28.9%, wasps 11.3%, beetles 7.8%, lepidopterans 3.6% and ants 3.2% (electronic supplementary material, figures S12). *Bombus sandersoni* was our most abundant hymenopteran species, followed by the wasps, *D. arenaria* and *D. norvegicoides*, megachilid leafcutter bees, sweat bees in the genus *Lasioglossum*, the cuckoo bumblebee *B. flavidus,* and *B. ternarius*. The Jaccard similarity indices for our networks collected across 3 years ranged from 83% to 86%, indicating high network similarity. We evaluated sampling completeness using the Chao 2 estimator which indicated that we sampled 85% of the plants and 59% of the flower visitor types in the community.

### (b) Floral reflectance spectra

**Figure 1.**
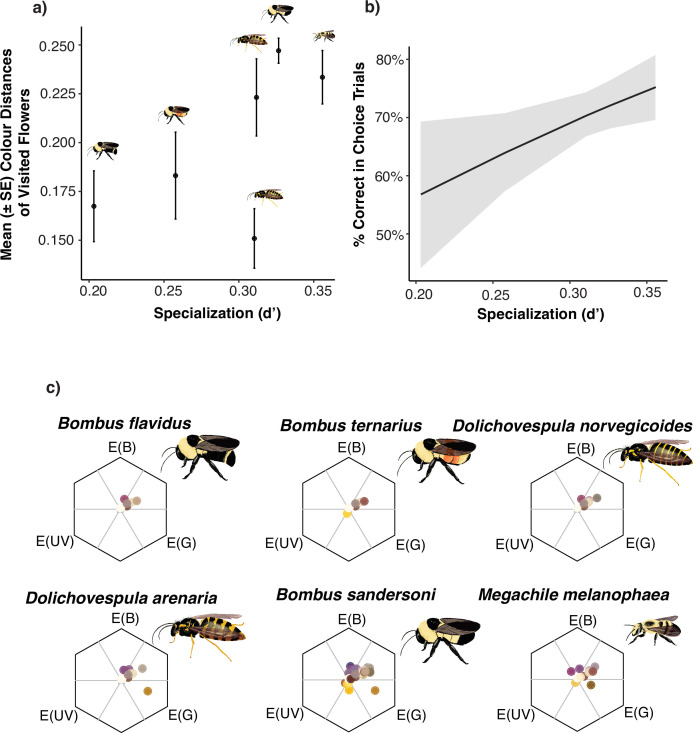
(a) The level of specialization of each tested insect species in the plant–pollination network and the mean chromatic contrast in honeybee colour vision space between the flowers they have been recorded visiting. More specialized insects (higher *d*′ values) visit a range of flowers with higher chromatic contrasts. *D. arenaria* is above *D. norvegicoides*. (b) Predicted probabilities from the binomial GLMM of the per cent correct choices insects made in the learning experiment according to their level of specialization in the network. (c) The flowers from which we recorded visits in the field by each of the species tested in the learning trails are plotted in honeybee colour vision space. Insect illustrations by Eva Ahn.

We measured the reflectance spectra of the flower petals of 90 plant species present on Kent Island (including 16 species we never had observed an insect visit and therefore are not in the network), and the two paper stimuli used for our colour learning experiment (electronic supplementary material, figure S3). The stimuli used in the learning experiment did not differ in their chromaticity distances from the flowers in the plant community (two-sample *t*‐test; *t* = −0.025, d.f. = 178, *p* = 0.98), i.e. one stimulus was not more similar to the colours of flowers available in the community than the other. There was a positive relationship between the level of specialization in the network (*d*′) of the hymenopteran species we tested in our associative conditioning experiment (electronic supplementary material, figure S4) and chromaticity distances of flowers visited by those species (linear model: *p* = 0.002, *R*^2^ = 0.0116; figure 1).

### (c) Colour learning experiment

Preliminary analysis of the complete generalized mixed-effect model showed no effect of the interaction between species and CS+ colour (binomial GLMM; *χ*^2^ = 6.93, d.f. = 5, *p* = 0.23) or species and trial number (*χ*^2^ = 1.21, d.f. = 5, *p* = 0.94), therefore these interaction terms were dropped from the final model which included trial number, CS+ colour and species. In the final model, there was an effect of trial number (binomial GLMM; *χ*^2^ = 9.24, d.f. = 1, *p* = 0.0024; electronic supplementary material, figure S5), with 64% of insects making a correct choice on the first trial up to 72% of insects making a correct choice on the fifth trial. There was also an effect of species (*χ*^2^ = 16.71, d.f. = 5, *p* = 0.0051) and *post hoc* comparisons showed *B. sandersoni* selected the CS+ over the CS− more often than *B. ternarius* (z = 2.91, *p* = 0.042), but no other species comparisons were statistically significant (*p* > 0.05). There was an effect of CS+ colour (*χ*^2^ = 7.19, d.f. = 1, *p* = 0.0073), with an average of 62% correct choices when the rewarded colour was blue and an average of 72% correct choices when the rewarded colour was yellow. In separate analyses, we ran the same model except including *d*′ values for each insect species from the plant–pollinator network instead of the species name. Correct choices were positively affected by *d*′ (*χ*^2^ = 5.19, d.f. = 1, *p* = 0.022), such that insect species with higher levels of specialization in our network performed better in our learning trials (Figure 1).

## 4. Discussion

We demonstrate differences in colour learning abilities of flower visitors that are associated with the level of specialization in the plant–pollinator network (*d*′) such that species with higher *d*′ values (more specialized in our network) performed better at a colour learning task than species with lower *d*′ values (more generalized in our network). The species with higher *d*′ values visited flowers with greater chromaticity distances than the insects with lower *d*′ values, which visited a narrower range of floral colours. We propose that there may be a feedback loop in which colour learning abilities enable pollinating insects to be efficiently selective about which plants to visit, and if these plants are of distinct colours, this may in turn strengthen selection for colour learning abilities. In contrast, in more generalist insects, selection of plants may be driven by how detectable plants are to the insect’s sensory system or sensory biases for particular colours, resulting in visiting a group of plants that are more similar in colour. Theory has proposed that species with intermediate degrees of generalization should have higher cognitive abilities than true specialists or true generalists [21]. The species in our study had *d*′ values ranging from 0.20 to 0.35, indicating fairly generalist species, whereas a true specialist would have a *d*′ value approaching 1. Within our range of generalist flower visitors, however, our finding of better performance in an associative conditioning task in species that are more specialized support the theory predicting higher cognitive abilities as insects move towards intermediate levels of specialization [21]. It is also important to note that in some bee species considered generalists at the species level, there are high levels of individual specialization [23–25], likely due to learning of flower types. We have assessed the level of specialization at the species level within the context of the Kent Island plant community. An ideal follow-up study would track consecutive flower visits for individual bees in the field [43] to determine levels of individual specialization for each species.

Differences in learning abilities could be associated with selective pressures on aspects of life-history aside from foraging. One proposed factor has been sociality. The social brain hypothesis suggests that sociality selects for cognitive abilities to remember individuals, which may result in better performance on other learning tasks [44]. One study, for example, showed that the solitary species of carpenter bee, *Xylocopa virginica*, is inferior at colour learning tasks to the social bumblebee, *Bombus bimaculatus* [45]. Another study, however, comparing bumblebees of social and parasitic species found that queens of the bumblebee *Bombus vosnesenskii* perform similarly to the queens of the parasitic cuckoo bumblebee *Bombus insularis* [46]. In our study, we tested the learning abilities of two social bumblebees: *B. sandersoni* and *B. ternarius*, a brood-parasitic cuckoo bumblebee, *B. flavidus,* which lays its eggs in the nests of other bumblebees and therefore does not live in social groups of its own species, two social wasp species, *D. arenaria* and *D. norvegicoides*, and a solitary leafcutter bee *M. melanophaea*. The only species differences we saw in learning were between social bumblebees: *B. sandersoni* and *B. ternarius*, therefore, our data do not support sociality impacting learning abilities.

Biases for certain colours are well-documented to influence learning in bees. In this study, as in many others, bees are trained to distinguish between colour stimuli, but there is an effect of which colour is rewarded on the learning outcome [12,47–50]. We found better performance when the CS+ colour was yellow, and of particular interest, this effect persisted across distantly related species. A preference for yellow has also been demonstrated in a similar experiment with wild-caught bumblebees in Western North America [46]. One possible explanation we considered was that our yellow stimulus was more similar to the colours of flowers available on Kent Island than our blue stimulus. However, we compared the chromatic contrasts and did not find differences between chromatic contrasts of the Kent Island flowers in comparison to the yellow stimulus versus the Kent Island flowers in comparison to the blue stimulus. Another possibility is that yellow flowers are more rewarding on Kent Island, as has been demonstrated for violet and blue flowers in Europe [10,51]. This is worthy of further investigation.

We used FMPER, an established protocol for field collection and testing of colour learning in pollinators [40,41,46], that has the benefit of enabling comparisons across diverse pollinator groups that are not available commercially or are difficult to rear in the lab [52]. Insects are tested in 50 ml tubes in this protocol, which is not physically constraining for any of our insects (i.e. they have plenty of room to turn around), but a potential downside to this method is that captivity stress may have different effects on insect learning depending on species. Another issue with this protocol is wild-caught adult insects are not naive, but have varied experiences that are likely to inform their decisionmaking in the test context. This is a downside that highlights the importance of balancing treatments as to which colours are associated with rewards as prior experience may influence colour preferences. With commercially available or lab-reared bumblebees, it is possible to conduct free-flying choice assays in the lab [48,53–57]. The free-flying nature of these assays likely more closely resemble learning and decision-making in wild bees. However, these assays usually require days of pre-training to encourage even tractable species like bumblebees to forage in experimental arenas, which is not possible unless colonies of the insects can be maintained in the lab. It would be worthwhile to use commercially available or lab-reared species to compare learning performance in FMPER protocols and performance in free-flying assays.

The ability to associate floral colours with rewards may be driving floral choices in the field. This would explain why more selective insects visit a broader range of flower colours and why they perform better in choice learning trials. Our network analysis classifies the insect species according to their degree of generalism, and our species are quite generalist. It may be, as is the case for many bumblebees [24], that these generalist insects show individual specialization through learning. In our community, the more generalist insects were visiting flowers that were more similar in colour than the more specialized insects. One possible explanation for these results is that within the more selective of these species, individuals may be specializing in flowers of particular colours aided by better colour learning abilities. The less selective species, in contrast, are visiting flowers that are more similar in colour, and may therefore have less individual specialization and lower colour learning abilities. Colour is only one of the floral traits that is driving insect decision-making. In particular, plant–pollinator network structure can be influenced by floral volatiles [14,58,59], plant phenology [60,61] and nectar quality and chemistry [62,63]. These factors are likely structuring the Kent Island network as well. Future research should investigate these other plant traits and link individual colour learning abilities in the lab with tracking of individuals as they visit consecutive flowers in the field, to determine if bee species that learn better in the laboratory tests show higher levels of individual specialization in the field.

## Data Availability

The datasets and R code for this article are available from the Dryad Digital Repository [64]. Supplementary material is available online [65].
